# Analysis of a Scenario for Chaotic Quantal Slowing Down of Inspiration

**DOI:** 10.1186/2190-8567-3-18

**Published:** 2013-09-16

**Authors:** C Baesens, RS MacKay

**Affiliations:** 1 Mathematics Institute, University of Warwick, Coventry, CV4 7AL, UK

## Abstract

On exposure to opiates, preparations from rat brain stems have been observed to continue to produce regular expiratory signals, but to fail to produce some inspiratory signals. The numbers of expirations between two successive inspirations form an apparently random sequence. Here, we propose an explanation based on the qualitative theory of dynamical systems. A relatively simple scenario for the dynamics of interaction between the generators of expiratory and inspiratory signals produces pseudo-random behaviour of the type observed.

## 1 Introduction

**Fig. 1:**
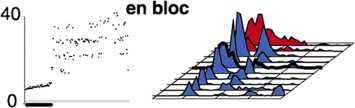
Sequential plot and histograms of inspiratory period for an en bloc in vitro preparation of rat brain stem before and after treatment with an opiate agonist, showing quantal slowing down of inspiration (reproduced with permission from [2]). *The horizontal axis* on the sequential plot is “cycle number” (meaning number of inspirations since a start time), *the vertical axis* is time between successive inspirations in seconds. *The horizontal axis* for the histograms is inspiration period and the histograms are plotted for ten different experiments. When the potassium concentration was increased in two experiments (the last two histograms, in *red*), the slowing of the inspiratory period was no longer quantised

Feldman et al. (e.g. the review [1]) observed that neonatal rat brain stems in vitro exposed to opiates continue to produce regular expiratory signals but fail to produce some inspiratory signals. The times between two successive inspirations form an apparently random sequence of multiples of the average expiratory period (see Fig. 1). The same has been seen in vivo [3]. 

Here, we investigate the possibility of an explanation based on the qualitative theory of dynamical systems: A relatively simple scenario for the dynamics of interaction between the generators of expiratory and inspiratory signals produces pseudo-random behaviour of the type observed.

Alternative explanations have been proposed in [4,5]. The first proposes a model with two physiologically plausible lumped oscillators; on exposure to opiates, they propose that the coupling becomes stochastic in time (one has to read the supporting information to [4] to find this), but we can generate the observed behaviour with deterministic chaos, which we consider more satisfactory than invoking stochastic coupling. The second simulates a large network of neurons (81 for expiration, 81 for inspiration), with random coupling, which may well be more realistic than just two, but their parameters are such that most oscillators in the same group fire together; indeed they call their model a “dual oscillator”, and it seems to us more simple to gain conceptual understanding by considering just two oscillators and to see whether there are dynamically plausible perturbations which could produce the observed effects. 

### 1.1 Normal Situation

Following [1], we assume that the several hundred neurons involved in generating breathing rhythm can be reduced to a system of two coupled oscillators. We assume the instantaneous state can be described by a pair (θ,ϕ) of phases (angles on a circle). When *θ* passes through a certain value $\theta_{0}$, a signal for expiration is generated; when *ϕ* passes through a certain value $\phi_{0}$, a signal for inspiration is generated. Compare “limit cycle” models, e.g. [6]. 

Evidence for two coupled but anatomically distinct rhythm generators is given in [2,3] (see also the recent review [7]). 

Under normal operation, the dynamics has an attracting 2-torus, containing an attracting periodic orbit of type (1,1) (one revolution in each of *θ* and *ϕ* per period), which crosses $\theta =\theta_{0}$ and $\phi =\phi_{0}$ once per period, giving alternating inspiration and expiration.

**Fig. 2:**
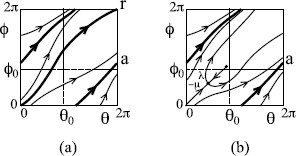
Examples of flows on a two-torus: **a** a Poincaré flow, **b** a Cherry flow

The flow on the 2-torus could be a “Poincaré flow” or a “Cherry flow”. A *Poincaré flow* is one with a “global cross-section”, a closed surface of codimension 1 (thus a circle in this case) transverse to the flow such that every trajectory cuts it in forward and backward time, as in Fig. 2(a) where we will choose {θ=0} as global cross section. In particular, it has no equilibria. A *Cherry flow* has at least two equilibria and (up to choice of direction of time) has a transverse circle for which the orbits of all points return except those which converge to a saddle; an example is shown in Fig. 2(b) (where the transverse circle can be taken to be θ=0 again). 

We consider both cases. The Poincaré case is the relevant one if the expiratory oscillator is sufficiently strong that it is affected little by the inspiratory oscillator; the observed quantisation of inspiratory period may be taken to support this. On the other hand, there must in normal operation be some mutual inhibition to prevent attempted simultaneous inspiration and expiration. It could be achieved by Poincaré flow if the attracting periodic orbit avoids the intersection $\left(\theta_{0},\phi_{0}\right)$ of the two threshold circles as in Fig. 2(a), but a stronger way would be a Cherry flow with $\left(\theta_{0},\phi_{0}\right)$ near the repelling equilibrium, as in Fig. 2(b).

**Fig. 3:**
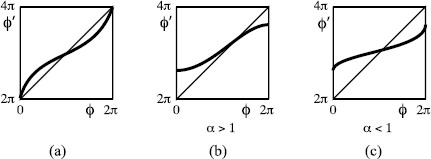
Sketch return maps for the flows of Fig. 2: **a** Poincaré flow, **b** Cherry flow with attracting saddle, **c** Cherry flow with repelling saddle

To study a flow, it can often be useful to take a “surface of section” *Σ*, a closed codimension-one surface (so circle in our case) transverse to the flow, and consider the return map *f* to it, given by following the flow to the first return (if any) with *Σ*. For a Poincaré flow, the return map is continuous and increasing and has “degree one”, meaning that on traversing *Σ* once, the image traverses *Σ* once in the same direction. A sketch of the return map for the flow of Fig. 2(a) to the section θ=0 (modulo 2*π*) is shown in Fig. 3(a). 

For the Cherry flow of Fig. 2(b), an appropriate surface of section is again the set θ=0. The return map then has the form of an increasing function with a jump at the image of the intersection of the stable manifold of the saddle with *Σ* as shown in Fig. 3(b) and (c). We choose to put the origin of *Σ* at the intersection with the stable manifold of the saddle so the jump corresponds to $\phi^{\prime }(2\pi )<\phi^{\prime }(0)+2\pi$. Since *Σ* is a circle, the point ϕ=2π represents the same point as ϕ=0, and we obtain the whole circle by taking the interval [0,2π). Although the return map is discontinuous, we consider the jump to be upward so as to make the resulting map have degree one.

The slopes of the return map for a Cherry flow at the ends of the discontinuity depend on the “exponent” α=μ/λ of the saddle, where the eigenvalues are labelled λ>0 and −μ<0. If α>1, the slopes are zero; if α<1, the slopes are infinite. More precisely, near the discontinuity (which we have agreed to put at 0), $f(x)∼A_{\pm }\pm C_{\pm }{\left|x\right|}^{\alpha }$ for *x* small positive and negative, respectively, some constants $A_{-}<A_{+}$, $C_{\pm }>0$ (we ignore the special case α=1, which typically involves a logarithmic correction). These are illustrated in Fig. 3(b) and (c).

### 1.2 Effect of Opiate Exposure

The effect of exposure to opiates is described in the experimental literature as suppressing the activity of the inspiratory oscillator, thus slowing it down in some way.

If there remains an attracting two-torus, then the winding ratio could cease to be locked to 1:1, but all trajectories would still have a common winding ratio. In particular, there would not be trajectories carrying out pseudo-random sequences of rotations. The sequence of numbers of expirations between successive inspirations would be a “rotation sequence”. This has a recursive definition. Firstly, there is an integer $n_{1}$ such that the numbers of expirations between successive inspirations are all either $n_{1}$ or $n_{1}+1$. Secondly, either the $n_{1}$ or the $n_{1}+1$ occur in singletons. Thirdly, the sequence of numbers of the other between these singletons forms a rotation sequence again. For example, EIEEIEIEEIEIEI is part of a rotation sequence, whereas EIEEIEEIEIEIEI is not. Rotation sequences are very special sequences and the results of Fig. 1 speak strongly against such sequences since it shows variation from 2 to 5 expirations between successive inspirations, contradicting the $n_{1}/n_{1}+1$ rule.

**Fig. 4:**
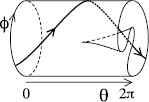
Insertion of a fold into the dynamics

Thus, we propose that the principal effect of opiate exposure is to make the attractor fold on itself as *θ* increases from generation of one expiratory signal to the next, as sketched in Fig. 4. As a result, increasing the initial value of *ϕ* at a given value of *θ* does not necessarily lead to increase of the value of *ϕ* after *θ* has increased by 2*π*. The resulting attractor may well be fractal in a transverse direction, but we assume that there is a strong stable foliation (e.g. [8]) whose leaves can be labelled by *θ* and *ϕ*. This assumption is probably false in detail, but we believe it will give the right idea (cf. much modelling of continuous-time systems by non-invertible maps, e.g. [9]). 

Such folded attractors with strong stable foliation can easily produce deterministic chaos of the form of pseudo-random sequences of rotations, as we shall now describe.

## 2 Analysis of the Scenario

The result of our proposal for the effect of opiate exposure is that *θ* continues to increase regularly in time, but *ϕ* may behave in a more complicated manner. Its evolution is most simply studied by considering the return map to the surface of section *Σ*. This map *f* is the composition of an increasing degree-one map, either continuous or having an upward jump discontinuity (as in Fig. 3), followed by a continuous degree-one circle map having one decreasing interval (resulting from the perturbation with a fold) that we denote (a,b).

**Fig. 5:**
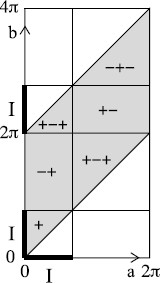
Two-parameter plane showing qualitative form of circle map for perturbed Cherry flow as the ends of the decreasing interval (a,b) move relative to the image *I* of the discontinuity. For this figure only, we put the origin at the left end of the interval *I*. Note that (a,b) is restricted to the range 0<a<2π, a<b<a+2π

Let us call the resulting class of maps *C*. In the Poincaré case, they are bimodal continuous, degree-one circle maps. In the Cherry case, let us denote by *I* the interval between the two branches of unstable manifold on θ=2π. Then there are various qualitative forms that maps f∈C can take, according to the disposition of the decreasing interval (a,b) with respect to the interval *I*, as indicated in Fig. 5. The regions are labelled by the sequence of signs of slope of *f* between 0 and 2*π*. 

The slopes of *f* at 0 and 2*π* are determined by the exponent of the saddle. If α>1, the slopes are 0; if α<1, they are infinite, except possibly in the special case that one end of *I* is *a* or *b*, when the outcome depends on how 1/α compares with the exponent of the fold (which is generically 2).

A key feature of an orbit of a degree-one circle map is its “rotation number”. This is the average rate (if it exists) at which the iterates $f^{n}(\theta )$, $n\in Z_{+}$, rotate around the circle. In our context, it gives the average ratio of numbers of inspirations to expirations. For a continuous monotone degree-one map of the circle, Poincaré proved that the limit exists and every orbit has the same rotation number. If monotonicity is broken, however, there is the possibility of more than one rotation number and even of orbits for which the average does not exist. More importantly, in this situation, there is the possibility of orbits with pseudo-random sequences of rotations per iteration. We will formalise this behaviour later in this section under the term “rotational chaos”.

For continuous bimodal circle maps, there are some nice results, such as that the set of possible rotation numbers is either a single point or a closed interval, and that existence of two periodic orbits with different rotation numbers implies existence of an invariant subset with pseudo-random sequences of rotations between those of the two periodic orbits [9]. Another result is based on the concept of a badly ordered invariant set. An invariant set for a degree one circle map is called *badly ordered* if it contains three points in clockwise order whose images are not in clockwise order. Any finite badly ordered invariant set implies similar rotational chaos [10]. We will give an example of a bimodal map for which there is rotational chaos with rotation interval $\left[\frac{1}{5},\frac{1}{2}\right]$.

Turning now to the case of discontinuous maps, maps of the form of region + were studied by [11-14]. As long as the jump discontinuity is upward, there is a unique rotation number, but for many of those with downward jump, the set of rotation numbers was shown to be an interval rather than a single point, points for which the rotation number does not exist were found and rotational chaos was found. We will give an example of a map in region + with rotational chaos, but the rotational chaos is attracting only when the saddle is repelling; otherwise, the rotational chaos is repelling and most trajectories go to a periodic orbit. 

More promising is the region −+. Here, we make examples with attracting rotational chaos even in the case of an attracting saddle.

We will give the examples first and then some general treatment.

### 2.1 Continuous Bimodal Examples

Consider continuous degree-one circle maps with a single decreasing interval.

**Fig. 6:**
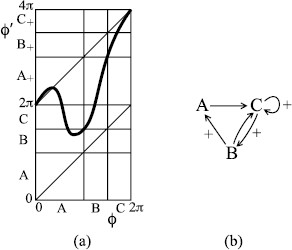
**a** A bimodal map possessing rotational chaos between rotation numbers $\frac{1}{2}$ and 1; **b** associated transition graph

Suppose the return map of Fig. 3(a) is deformed by the fold into Fig. 6(a). It still has a fixed point of rotation number 1 at ϕ=0 and another inside the interval *A* but now has also a period-2 orbit of rotation number $\frac{1}{2}$ (its points are at the *AB* and *BC* boundaries). The fixed point and period-2 points partition the circle into three arcs *A*, *B*, *C*. 

**Definition** Say a 1D map *f* maps an interval *Aover* an interval *B* if *f* is continuous on *A* and there is a subinterval $A^{\prime }$ of *A* such that $f(A^{\prime })=B$ (cf. [15]). 

This definition implies that the image of *A* covers *B* and possibly more, but is more precise, allowing application of the intermediate value theorem to deduce that if *f* maps *A* over *B*, *B* over *C*, etc., then there is a point x∈A such that f(x)∈B, $f^{2}(x)\in C$, etc.

The arcs *A*, *B*, *C* are mapped over each other as indicated in the transition graph of Fig. 6(b). Here, we have introduced notation _+_ to show when trajectories pass 2*π*, thus making one revolution; it can be taken as indicating inspiration. The beauty of the notion of “mapping over” is that every path in the graph is taken by some trajectory. In particular, pseudo-random paths in the graph give trajectories generating any sequence of inspiration or not between successive expirations. We call it “rotational chaos”.

The downside of examples such as this is that the set exhibiting rotational chaos might not be attracting; most trajectories might do something less interesting, like converging to the attracting fixed point in the interior of arc *A*, which corresponds to alternating inspiration and expiration.

**Fig. 7:**
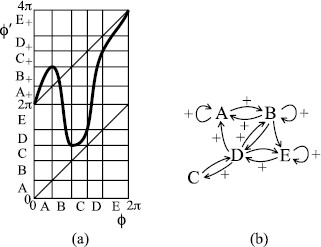
**a** A bimodal example with the orbits of the critical points landing on an unstable period-2 orbit; **b** associated transition graph

To make examples where typical trajectories exhibit rotational chaos, one way is to suppose the map has negative Schwarzian derivative (a relatively mild condition which can be written as ${\left(1/{\left(\log|f^{\prime }|\right)}^{\prime }\right)}^{\prime }>-\frac{1}{2}$) and to oblige the orbits of the critical points (turning points) of the map to land on unstable periodic orbits. The point is that, for a map with negative Schwarzian derivative, every attracting periodic orbit attracts the orbit of at least one critical point, so if the orbits of the critical points go to unstable periodic orbits there can be no attracting periodic orbits. Furthermore, with respect to the finite partition created by the orbits of the critical points, the dynamics are equivalent to a type of stochastic process called Gibbsian. Assuming the transition graph has a single communicating component (a communicating component of a directed graph is a maximal subset of the vertices such that for all pairs *A*, *B* it is possible to go from *A* to *B* and back to *A*), the process has unique invariant probability distribution, given by an $L^{1}$ density *ρ* on the circle satisfying the Perron–Frobenius equation

$$\rho (x)=\sum_{y\in f^{-1}(x)}\frac{\rho (y)}{\left|f^{\prime }(y)\right|}$$

and the normalisation condition ∫ρ(x)dx=1. The theory is highly technical so we restrict ourselves to giving one recent reference [16]. If the transition graph allows more than one rotation number, then this probability distribution exhibits rotational chaos. Thus, we modify Fig. 6 to Fig. 7. Note that the associated transition graph has rotational chaos with all rotation numbers between $\frac{1}{2}$ and 1. 

**Fig. 8:**
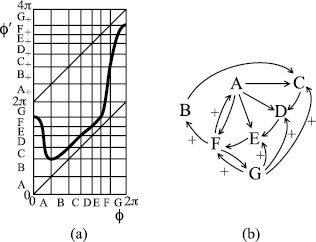
**a** A bimodal example with rotation interval $\left[\frac{1}{5},\frac{1}{2}\right]$; **b** transition graph

The same idea motivates the example of Fig. 8, which has rotational chaos between rotation numbers $\frac{1}{5}$ and $\frac{1}{2}$, to match the results of Fig. 1. 

The condition that the critical points be pre-periodic is strong, but similar chaotic properties with an absolutely continuous probability distribution can be derived if the orbits of the critical points do not come back too close too soon in a suitable sense. The theory of this is even more technical, beginning with the Collet and Eckmann condition (see [17] for a survey), but the condition holds for a set of parameters of nearly full measure near the cases with pre-periodic critical points. 

### 2.2 Discontinuous Type + Examples

Now we turn to discontinuous circle maps.

**Fig. 9:**
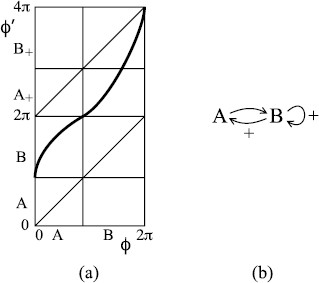
**a** Example of return map of type +; **b** associated transition graph

In region + of Fig. 5, the decreasing interval lies inside *I* and if the fold is strong enough it can make the discontinuity of *f* at 0 jump downward instead of upward. In the case of repelling saddle, a possible result is indicated in Fig. 9(a). Here, the circle is partitioned into two intervals *A*, *B* as shown. The interval *A* maps onto *B* and the interval *B* maps onto the union of $A_{+}$ and $B_{+}$. The resulting transition graph is shown in Fig. 9(b). Every path in this graph is taken by some orbit. In particular, the period-1 cycle $B\to B_{+}$ has rotation number 1, the 2-cycle $A\to B\to A_{+}$ has rotation number 1/2, and pseudo-random paths in the graph can be taken to generate any sequence of inspiration or not between successive expirations. 

The example is very particular in that f(2π)=4π and $f^{2}(0)=2\pi$ (just as we made the orbits of critical points of bimodal maps land on unstable periodic orbits). If these conditions are not met exactly, the dynamics can be more complicated to describe but may often imply similar pseudo-random behaviour. A complete theory of the dynamics has been given if the slope of *f* is everywhere greater than 1 (or under a weaker topologically expanding condition) [11-14], but we will content ourselves with presentation of examples. 

**Fig. 10:**
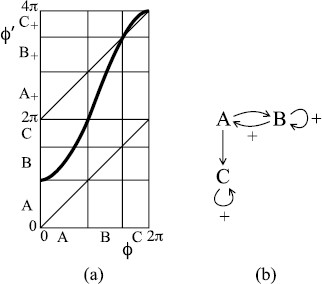
**a** Variant of Fig. 9 with attracting instead of repelling saddle; **b** associated transition graph

If we modify the example to correspond to an attracting rather than repelling saddle, we obtain Fig. 10, for which the same rotational chaos exists with the smaller interval B, but a transition from *A* to a third interval *C* is also possible and *C* is absorbing. Typical orbits in A∪B perform a chaotic transient of pseudo-random behaviour, but eventually exit to *C* and thereafter exhibit 1:1 behaviour, which is less relevant. 

### 2.3 Discontinuous Type −+ Examples

**Fig. 11:**
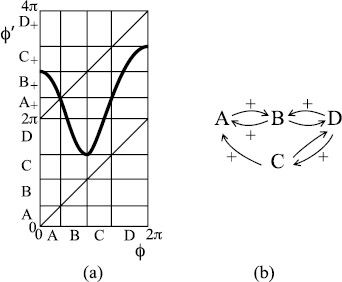
**a** Example of return map of type −+, **b** associated transition graph

In region −+, we can make examples like Fig. 11(a). With the given partition, we obtain the graph of allowed transitions in Fig. 11(b). There is 1:1 behaviour for the cycle $A\to_{+}B\to_{+}A$ and 1:2 behaviour for the cycles $B\to D\to_{+}B$ and $C\to D\to_{+}C$. There is pseudo-random rotational behaviour, with missing inspirations whenever B→D or C→D. 

**Fig. 12:**
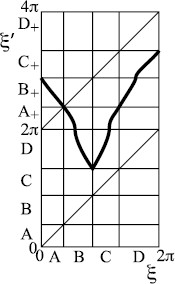
The map of Fig. 11 expressed in a new coordinate *ξ* chosen to make the slope everywhere greater than 1

One may worry that because of the regions with slope near zero there might be periodic attractors and the rotational chaos might be repelling and, therefore, only transiently visible. But for this example one can make coordinate changes near each of the points of zero slope to make the slope greater than 1 in absolute value, at the expense only of introducing infinite slope at the pre-images of the critical points as in Fig. 12. 

Although drawn for the case of attracting saddle, the example works equally well with repelling saddle.

**Fig. 13:**
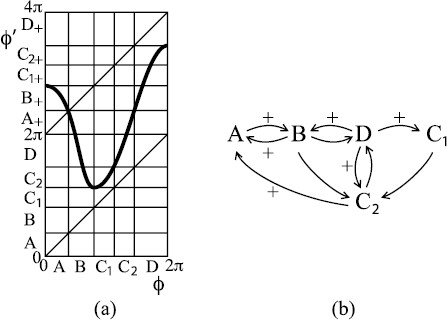
**a** Modification of Fig. 11, **b** associated transition graph

We can make a variant with the additional possibility of missing two successive inspirations, as indicated in Fig. 13. The construction can be extended to skip any number of inspirations. 

**Fig. 14:**
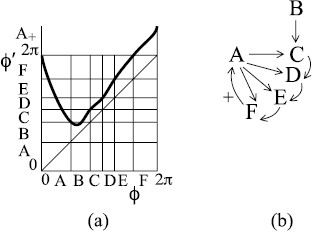
Example of type −+ with rotation interval $\left[\frac{1}{5},\frac{1}{2}\right]$: **a** map, **b** associated transition graph

To make an example which produces behaviour like that of Fig. 1, we take repelling saddle and a map of type −+ as in Fig. 14. It exhibits rotation numbers from 1/5 to 1/2, hence number of expirations between successive inspirations from 2 to 5. Note that *B* maps over *C*, but there is no route back to *B* from the rest. All orbits eventually leave *B*, so we could consider the map on its complement. 

### 2.4 Rotational Chaos

To make precise statements about the behaviour exhibited by the examples, we introduce a definition.

**Definition** A circle map exhibits *rotational chaos* if it has an invariant subset with a semi-conjugacy to a topological Markov chain whose “translation co-cycle” is non-trivial.

We have to explain the various terms in this definition.

A *topological Markov chain* (TMC) is a discrete-time dynamical system based on a finite directed graph with edge set *Γ*, whose state space is the set $P_{\Gamma }$ of doubly infinite paths γ:Z→Γ endowed with the product topology, and whose map is the shift $\sigma :P_{\Gamma }\to P_{\Gamma }$, $\sigma {\left(\gamma \right)}_{n}=\gamma_{n+1}$. It is *irreducible* if for every two nodes *x*, *y* in the graph there is a path from *x* to *y*.

A map f:F→F is *semi-conjugate* to g:G→G if there is a continuous surjection h:F→G such that hf=gh.

A *co-cycle* for a map f:F→F is a continuous function k:F×N→R such that $k(x,n+m)=k(x,n)+k(f^{n}x,m)$ for all x∈F, n,m∈N. It is enough to specify k(x,1) for all *x*, but traditional to define it as above because the interest is in how k(x,n) grows with *n*.

We endow any TMC arising from a degree-1 circle map *f* with a *translation co-cycle*, defined by making a choice *S* of lifts to ℝ of the subsets representing the vertices of *Γ*, and a choice $\tilde{f}$ of lift of *f* (map $\tilde{f}:R\to R$ such that $\pi \tilde{f}=f\pi$, where π:R→R/2πZ is π(x)=x(mod 2πZ)) and letting k(x,1) be the integer such that for *x* represented by *γ*, then f(x)∈S+k(γ,1). If *k* depends on only $\gamma_{0}$ we say it is *elementary*.

A co-cycle *k* for *f* is *non-trivial* if there are no c∈R and continuous function *b* such that k(x,1)=c+b(fx)−b(x).

A *simple* cycle for a TMC is a closed path in the graph, which visits no vertex more than once.

**Theorem***An elementary co*-*cycle on an irreducible TMC is non*-*trivial iff there are two simple cycles with differing average*.

*Proof* If there are two cycles (simple or not) with differing averages *s*, *t*, let *N* be the least common multiple of their periods. Then k(x,N)=Ns for one and *Nt* for the other, whereas if the cocycle were trivial it would be *Nc* for both.

In the other direction, if all simple cycles have the same average, call it *c*, then the same holds for any periodic cycle because it can be broken into segments between repeated vertices which can be rearranged to make a sum of simple cycles, and the value of the co-cycle is simply the sum because it is elementary. Choose a reference vertex, let b=0 there and let $b=\sum_{i=1}^{n}k(\gamma_{i},1)-nc$ at the end of each path from the reference vertex. This defines *b* uniquely because if two paths end at the same vertex then close them back to the reference vertex by a common path to see that *b* was uniquely defined. Then k(γ,1)=c+b(σγ)−b(γ), so the co-cycle is trivial. □

**Corollary***If a degree*-*one circle mapfhas a set of intervals*$A_{j}$*which are mapped over each other according to an irreducible graphΓand the graph contains two simple cycles with differing average translation thenfshows rotational chaos*.

**Fig. 15:**
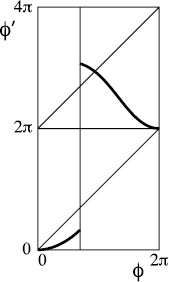
An example with periodic orbits of two different rotation numbers but no rotational chaos, because the left interval is invariant with rotation number 0, what does not stay in the right interval falls into the left, and what does stay in the right has rotation number 1

**Fig. 16:**
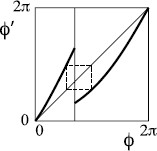
An example with a badly ordered period-2 orbit but no rotational chaos, because the interval [0,2π] is invariant

*Remarks* One might have thought that having two periodic orbits with different rotation numbers would suffice for rotational chaos (as is true in the continuous case), but Fig. 15 gives a counterexample in the discontinuous case. In the continuous case, any badly ordered finite set suffices for rotational chaos [10], but Fig. 16 shows that in the discontinuous case this does not even imply non-trivial rotation set. 

### 2.5 Quantisation of the Time Between Inspirations

We have proposed a scenario that naturally leads to pseudo-random sequences of numbers of expirations between successive inspirations, but it remains to explain why the time between inspirations is quantised.

Our scenario consists of two coupled oscillators whose dynamics generates an attractor of the form of a torus with a fold. The attractor may be fractal but we suppose that we can still define phases *θ* and *ϕ* on it, representing the phase of the expiratory and inspiratory oscillator, respectively.

The quantisation of the time between inspirations is most simply justified if the flow is a “skew-product”, meaning that the *θ* dynamics is unaffected by the *ϕ* dynamics, with $\dot{\theta }>0$ for all *θ*. Then the time from one expiration to the next is independent of what *ϕ* does, and the time between successive inspirations is closely determined by the number of times the trajectory winds in the *θ* direction for one revolution in *ϕ*. The skew-product case would give a Poincaré flow under normal operation.

The quantisation of the inspiration period is less easy to justify in the Cherry case. Indeed, if some of the pseudo-random trajectories pass close to the saddle, then they will be slowed down there and there is no reason for the time between inspirations to be close to a multiple of the average expiration period. Close to quantised behaviour might result, however, if the orbits come close to the saddle only rarely.

**Fig. 17:**
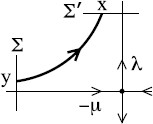
Orbit near a saddle point

For a saddle with eigenvalues −μ<0<λ and transverse sections *Σ*, $\Sigma^{\prime }$ to its stable and unstable manifolds, the time *τ* taken from *Σ* to $\Sigma^{\prime }$ is determined asymptotically by

$$y_{1}∼ye^{\lambda \tau }$$

for the orbit starting at *y* on *Σ* ($y_{1}$ a constant); see Fig. 17. So $y∼y_{1}e^{-\lambda \tau }$. So a probability density $\rho_{y}$ for *y* gives one $\rho_{\tau }$ for *τ* with

$$\rho_{\tau }=\rho_{y}|\frac{dy}{d\tau }|∼\rho_{y}\lambda y_{1}e^{-\lambda \tau }.$$

If

(1) $$\rho_{y}\leq Ky^{-\beta }\;\text{with }\beta <1$$

then

$$\rho_{\tau }≲\lambda Ke^{-\lambda (1-\beta )\tau },$$

which gives exponentially small probability of taking a long time to pass the saddle. 

So the question is whether (1) might be satisfied. There are results which give invariant probability with bounded density (thus β=0) for a one-dimensional map, but they generally require the map to be expanding and a bound on its second derivative, e.g. [18]. The former would appear to rule out attracting saddle and critical points, and the latter to rule out repelling saddle. Nevertheless, after suitable coordinate change or inducing on an appropriate subset, it can sometimes be possible to turn a map into one to which such results can be applied [19]. A classical example is for the map $f(x)=2-x^{2}$ on [−2,2] which has a critical point at x=0, the coordinate change x=−2cosθ, θ∈[0,π] turns it into the tent map with slope =±2. Suppose z=g(y) is a coordinate for which an invariant measure has bounded density $\rho_{z}\leq M$. Then

$$\rho_{y}(y)=\rho_{z}\left(g(y)\right)g^{\prime }(y).$$

Thus, $\rho_{y}(y)\leq Ky^{-\beta }$ if $g^{\prime }(y)\leq \frac{K}{M}y^{-\beta }$, which is a mild condition on *g*.

On the other hand, an advantage of our scenario is that it does not automatically quantise the time between inspirations. Increasing the potassium concentration destroys the quantisation, as remarked in Fig. 1, so one wants a model that retains this possibility, as our does.

## 3 Conclusion

We have given a possible dynamical systems explanation of the chaotic quantal slowing down of inspiration observed under the effects of opiates in rats.

In summary, we propose that the effect of opiates is to introduce a fold into the dynamics of two coupled oscillators. This can produce pseudo-random sequences of numbers of expirations between successive inspirations. We also showed it is plausible that the times between successive inspirations remain close to multiples of an average inter-expiration period.

It will be interesting to test this proposal physiologically. In particular, could one construct the return map from experimental results such as those used to make Fig. 1? If the phases are not directly observable, one could perhaps reconstruct something equivalent from the times between inspiration or expiration. We elaborate on this last idea in Appendix A, though it is a project which would deserve a paper in itself.

A simple data analysis technique that could reveal something of the “grammar” of the expiration/inspiration sequences is described in Appendix B.

## Appendix A

If *φ* is a $C^{1}$ flow on a manifold *M* of dimension *d* and Σ⊂M is a codimension-1 $C^{1}$ submanifold transverse to the flow, then the return-time function $\tau :\Sigma_{1}\to R_{+}$ is a $C^{1}$ function on the subset $\Sigma_{1}$ of *Σ* which returns. Denote the return map $f:\Sigma_{1}\to \Sigma$; it is also $C^{1}$. Then, given n≥1, define the map $\Phi :\Sigma_{n}\to R_{+}^{n}$ on the subset $\Sigma_{n}$ of *Σ* which returns at least *n* times, by $\Phi (x)=(\tau (x),\tau (f(x)),\ldots ,\tau (f^{n-1}(x)))$. For generic *φ*, *Σ*, the map *Φ* is a $C^{1}$ embedding of $\Sigma_{n}$ into $R_{+}^{n}$ if n≥2d−1, by analogy with Takens’ embedding theorem [20]. Thus, given the sequences of return times for some trajectories on an attractor *Λ* of *φ*, one can reconstruct Λ∩Σ up to $C^{1}$ diffeomorphism. Furthermore, one can reconstruct the return map *f* from the shift on the sequences: $\left(\tau_{1},\ldots ,\tau_{n}\right)$ determines a point on Λ∩Σ and $\left(\tau_{2},\ldots ,\tau_{n+1}\right)$ determines its image.

One could attempt to apply this to the breathing data by taking *Σ* to correspond to expiration, assume d=3 suffices, and hence n=5, assume that every orbit on the attractor returns to *Σ* infinitely often, and apply the method to the sequence of times between successive expirations, to deduce up to diffeomorphism what Λ∩Σ and the return map to it look like. In practice, however, this is unlikely to reveal much, as the time between successive expirations is close to constant.

One might propose instead to take *Σ* to correspond to inspiration, for which the return time is quite variable, but the submanifold corresponding to inspiration is unlikely to be transverse to the flow, as the phenomenon is that some approaches to inspiration fail to cross and have to wait for a later attempt.

If both the expiration and inspiration submanifolds (call them *E* and *I*) were transverse to the flow, one could contemplate an extension of the above idea to take the sequence of times between both inspiration and expiration events, adding symbols *I* or *E* to indicate which, e.g. $\ldots Et_{1}It_{2}Et_{3}Et_{4}I\ldots$ . Although this representation is discontinuous at E∩I, we are expecting *Λ* to avoid E∩I (simultaneous expiration and inspiration). If *Λ* does come close to E∩I, we could make multiple charts for *E* and *I* by eliminating events that follow in too short a time, but would then have to deal with the overlap of charts, which although feasible is messy. In any case, for the present application *I* is unlikely to be transverse to the flow so this would produce a discontinuous representation of E∩I, so we leave the discussion here.

## Appendix B

The idea is to map the observed sequences into a square as follows: Choose *λ* a little less than 1/2. Given a sequence $a_{0}a_{1}\ldots a_{t}\ldots a_{N}$ of *E* and *I*, for each *t*, map $a_{t}a_{t+1}\ldots$ to

$$f_{t}=(1-\lambda )\sum_{s\geq t,a_{s}=I}\lambda^{s-t}\in [0,1]$$

and $\ldots a_{t-2}a_{t-1}$ to

$$p_{t}=(1-\lambda )\sum_{s\leq t-1,a_{s}=I}\lambda^{t-1-s}\in [0,1].$$

We call $f_{t}$ the future and $p_{t}$ the past. Then plot the points $\left(p_{t},f_{t}\right)$ in the unit square, as *t* goes from *m* to N−m for some *m* such that $(1-\lambda )\lambda^{m}$ is not visible. If all sequences occur, then they will fill out a 2*D* middle (1−2λ) Cantor set. If only a rotation sequence occurs, then $f_{t}$ is a decreasing function of $p_{t}$ (if it is periodic then only finitely many points appear). For examples like Fig. 9, the sequences fill out a sub Cantor set, revealing the associated transition graph.

Such plots have been used to study the “pruning front” conjecture [21]. 

For an example of use of a related technique to reveal a grammar in sequences of positive integers, see [22]. 

## Competing Interests

The authors declare that they have no competing interests.

## Authors’ Contributions

The authors contributed equally to the work.
